# Supplementary material for: Interspecies conservation of organisation and function between nonhomologous regional centromeres
Source: Nat Commun. 2019 May 28;10:2343. doi: 10.1038/s41467-019-09824-4 (PMC6538654; doi:10.1038/s41467-019-09824-4)
Supplement: Supplementary file 1 — Supplementary Information [file 41467_2019_9824_MOESM1_ESM.pdf]

Interspecies conservation of organisation and function between nonhomologous regional centromeres

Tong et al.

a

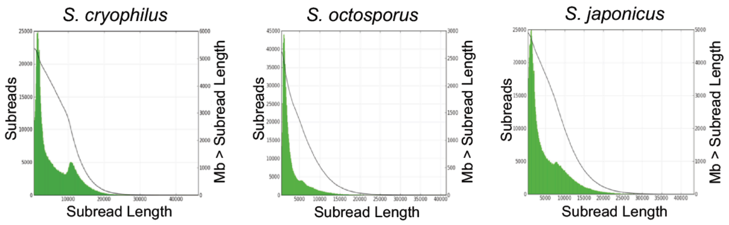

b

	S. cryophilus	*S. octosporus*	*S. japonicus*
Contig N50	2,797,701	3,794,048	1,051,694
Max contig length	4,890,599	4,519,984	2,096,611
Number of contigs	12	39	280
Sum of contig lengths	11,965,400	11,871,057	16,757,317
Published genome length (Broad)	11,589,478	11,678,700	11,813,213
Number of SMRT cells	11	11	15
PacBio reads N50	10,335	5,323	8,715
PacBio reads N90	16,558	12,893	16,805
PacBio Longest read	46,162	41,378	43,216

c

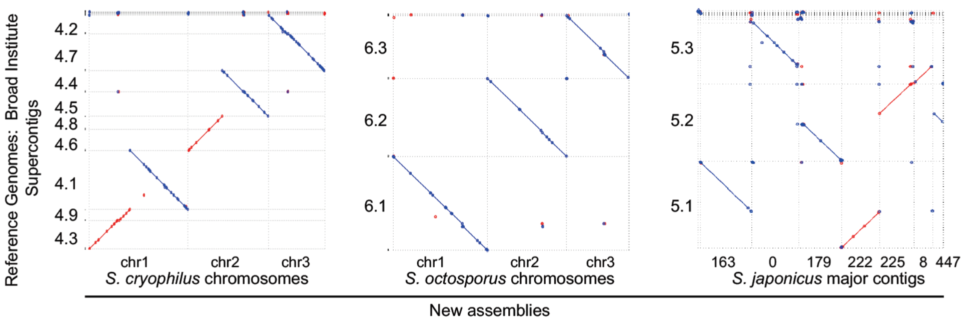

d

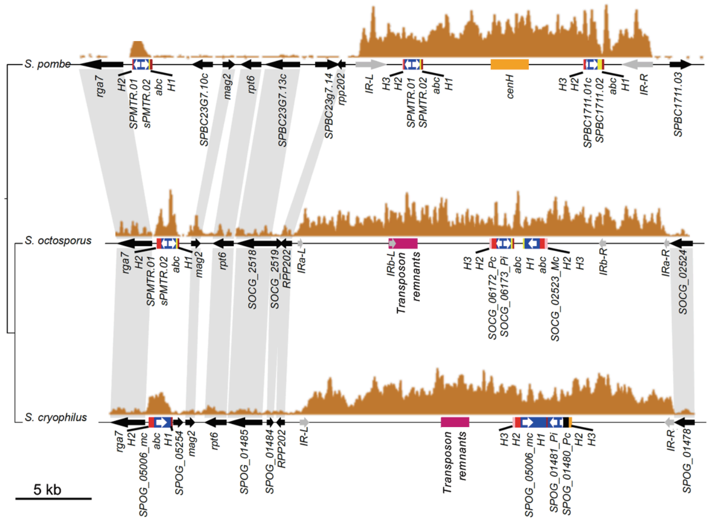

Supplementary Figure S1

Supplementary Figure 1: *S. octosporus* and *S. cryophilus* genome assembly statistics

a) Histograms of SMRT cell subread lengths (green) and the sum of subread length (black) for the indicated genomes.

b) Summary of PacBio subreads and final assemblies.

c) Dot plot comparison of new assemblies with previously-published assemblies for *S. octosporus*, *S. cryophilus* and *S. japonicus*[1]. A recent study reported joining the previously-published *S. cryophilus* supercontigs[1] into chromosome arm-sized assemblies and provided PCR-based support for juxtaposition of Sc9-Sc1, Sc5-Sc8 and Sc8-Sc6[2]. However, as centromere regions were not assembled in that study, linkage of chromosome arms could not be demonstrated[2].

d) Organisation of mating-type loci in *S. pombe*, *S. octosporus* and *S. cryophilus*[1,3]. ChIP-seq profiles for H3K9me2-heterochromatin (orange) are shown. Positions of mating-type loci (blue) and mating-type genes (white), mating-type associated repeat elements (H1: dark red; H2: red; H3: pink; abc: yellow); transposon remnants (pink), inverted IR repeats (grey) and other genes (black) are indicated. *cenH* region (orange) homologous to *S. pombe* centromeric *dg/dh* repeats is indicated. Grey shading indicates homologous genes between species.

Source data available: GEO: GSE112454.

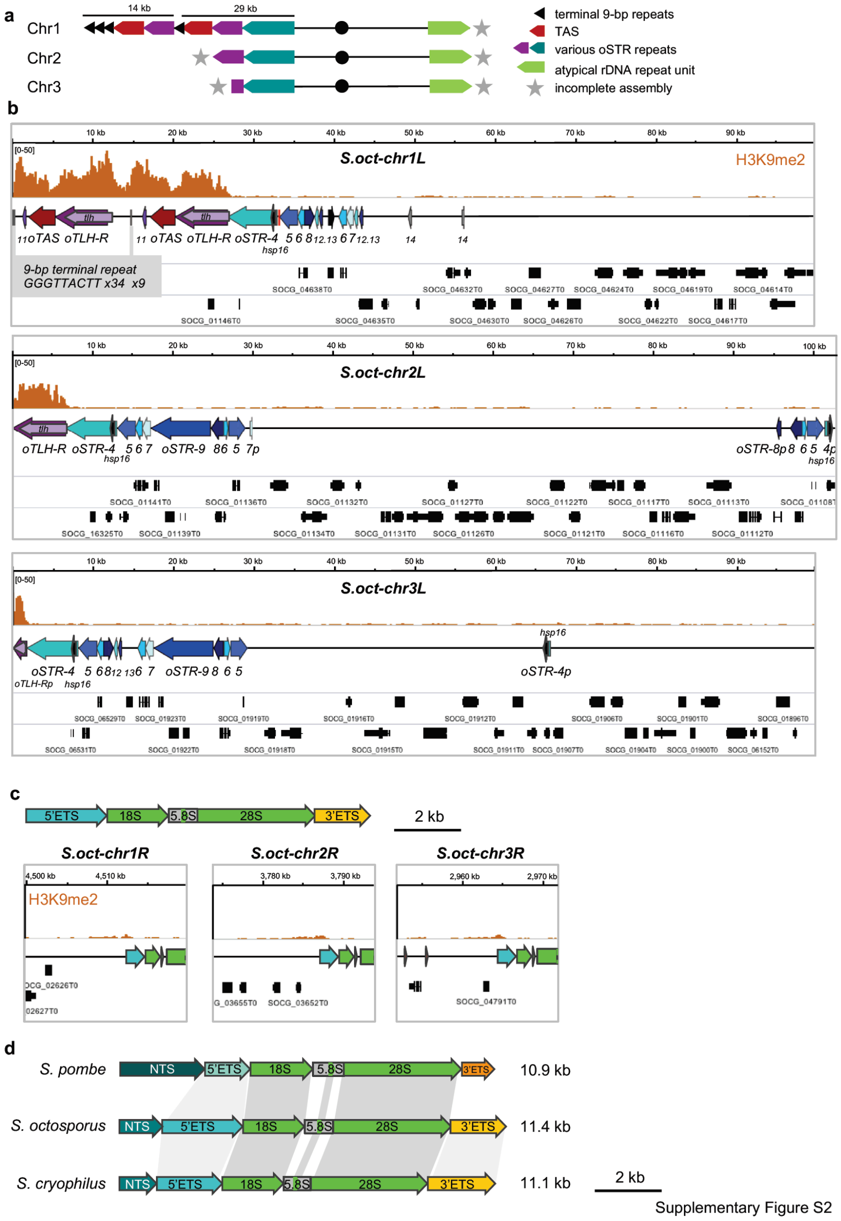

Supplementary Figure 2: Structure of *S. octosporus* chromosome ends

a) Overview of *S. octosporus* chromosomes, indicating organisation of subtelomeres. Multiple copies of terminal repeats (black arrows; GGGTTACTT) are detected at the end of chr1L (and internally). Combinations of subtelomeric repeats, including telomere-associated sequences (oTAS, dark red); RecQ type DNA helicase genes (*tlh*) and associated repeats (oTLH-R) and other subtelomeric repeats (oSTR-4 etc; turquoise); details in (b). A partial atypical rDNA repeat is detected at: chr1R, 2R and 3R (light green arrow). Due to repetitive nature of this region, assemblies are incomplete at all chromosome ends (denoted by grey star), except chr1L.

b) Details of terminal 100 kb of chr1L, chr2L and chr3L. Two copies of telomere-associated sequences (oTAS; dark red), and oTLH-R (containing RecQ type DNA helicase genes (*tlh*)) are present at chr1L, along with multiple copies of GGGTTACTT repeats. Numerous other subtelomeric repeats, designated oSTR-4 etc (blue/turquoise) are indicated, mostly by number designation only due to space constraints.

c) Top, structure of atypical rDNA repeat unit (lacking the full NTS seen in typical rDNA repeats, see (d). Atypical rDNA repeat units are present at centromere-proximal side of chromosome ends: chr1R, 2R, 3R. Due to repetitive nature of these regions, full assembly was not achieved and the number of rDNA repeat units present at each chromosome end is unknown. From ChIP input read counts the total number of rDNA repeat units is estimated to be approximately 150 copies.

d) Homology of rDNA repeat unit between *Schizosaccharomyces* species.
S. pombe, *S. cryophilus* and *S. octosporus* rDNA repeats are shown. *S. pombe* elements were previously defined[4]. Homology indicated by grey blocks: darker grey indicates higher homology (65%-92%). Source data available: GEO: GSE112454.

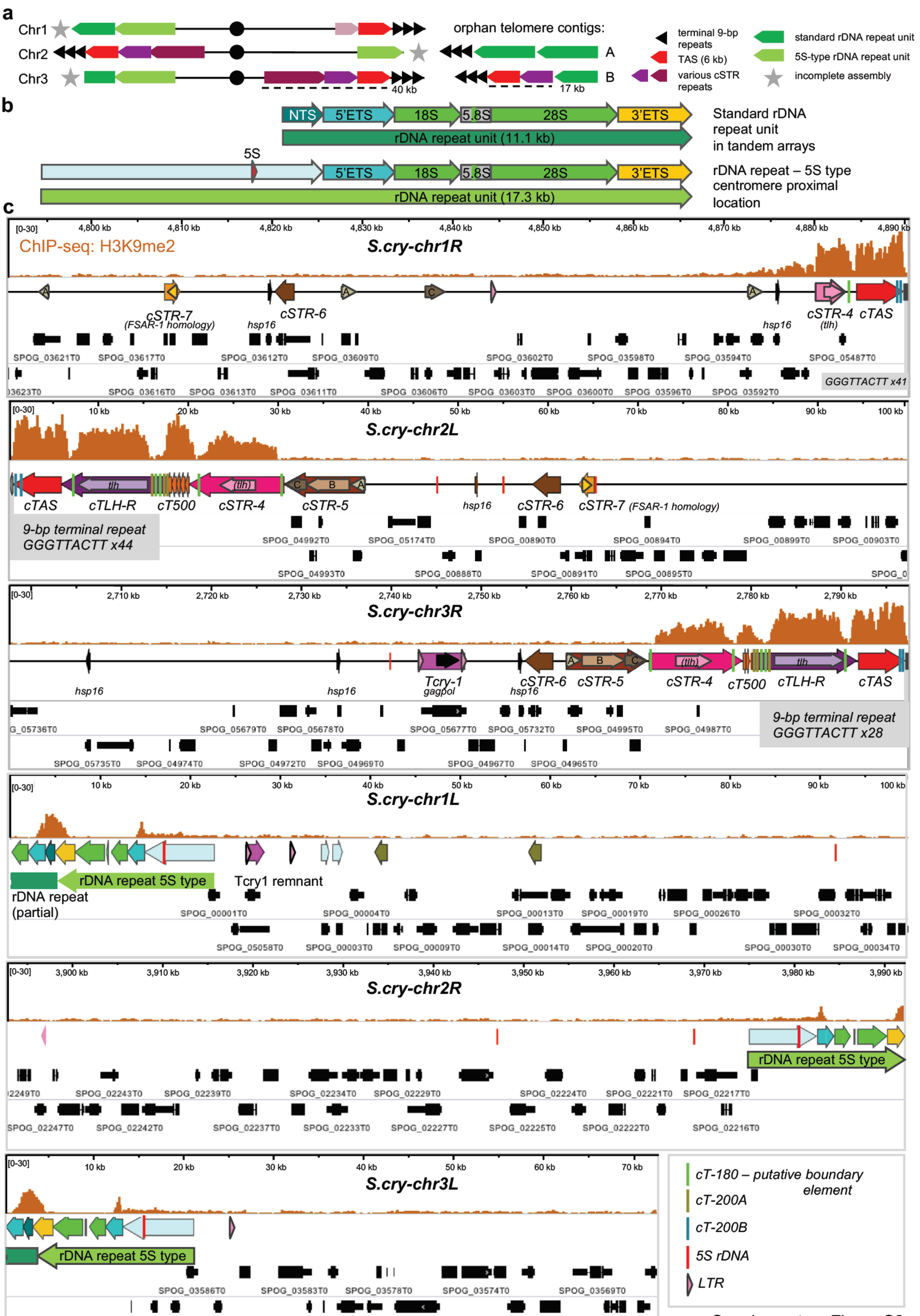

Supplementary Figure S3

Supplementary Figure 3: Structure of *S. cryophilus* chromosome ends

a) *Left*, overview of *S. cryophilus* chromosomes, indicating organisation of subtelomeres. Centromeres are indicated (black circles). Multiple copies of terminal repeats (black arrows; GGGTTACTT) are present at the ends of 1R, 2L and 3R, along with combinations of subtelomeric repeats, including telomere-associated sequences (cTAS, red); RecQ type DNA helicase genes (*tlh*) and associated repeats (cTLH-R) and other subtelomeric repeats (cSTR-4 etc; shades of pink/brown); details in c. rDNA repeats are located at: 1L, 2R and 3L. Centromere-proximal rDNA repeat is atypical (light green arrow) and associated with a 5S rDNA (details in b). Distal to that, assemblies of chromosome 1 and 3 indicate a partial standard rDNA repeat (no associated 5S rDNA; dark green). Due to repetitive nature of this region, assemblies are incomplete at 1L, 2R and 3L (denoted by grey star). *Middle*, two classes of terminal rDNA-containing contigs were also identified. Both types contain multiple copies of the terminal GGGTTACTT repeat. In class A these directly abut rDNA repeat units. In class B, cTAS and cTLH-R elements are located between the terminal repeats and rDNA repeat unit. A similar arrangement has recently been described for the rDNA-containing ends of *S. pombe* chromosome 3[5]. *Right*, key to repeat elements.

b) Two types of rDNA repeat are present in *S. cryophilus*. *Top*, standard repeat of 11.1 kb, present in tandem arrays. 28S, 18S and 5.8S genes are indicated (green), along with putative associated elements external transcribed spacers (5'ETS, turquoise; 3'ETS, yellow) and non-transcribed spacer (NTS, teal). *Bottom*, atypical rDNA repeat located at centromere-proximal location of all three rDNA-containing chromosome ends. In place of standard NTS element it is associated with a 7.5 kb repeat (pale blue) containing a 5S rDNA gene (red arrow).

c) *Upper 3 panels:* Subtelomere-repeat-containing chromosome ends are assembled in heterochromatin. Terminal 100 kb of arms 1R, 2L and 3R are shown. Location of repeat elements are indicated, along with positions of *hsp16* genes. Smaller repeat elements are indicated by vertical bars (see key, bottom right). H3K9me2 ChIP-seq profile is shown (orange). cT-180 repeats (green bars) coincide with deep troughs in H3K9me2 reads, suggesting that these elements could perform a boundary function. cTLH-R contains intact *tlh* genes, whereas cSTR-4 elements contain degraded copies of *tlh*. The partial cSTR-4 element has weak homology to cSTR-4 at 2L and 3R and a highly degraded copy of *tlh*. Location of genes indicated in black. The non-heterochromatic portion of the subtelomeres contain several paralogous genes, homologues of which are also found in the subtelomeric regions of *S. octosporus*. The intact retrotransposon *Tcry1* (magenta; LTRs, pink arrows) is located in the subtelomeric region of 3R.

Lower 3 panels: Chromosome ends with rDNA repeat units: terminal 100 kb of 1L, 2R, 3L shown. Feature colours as in a, b. H3K9me2 ChIP-seq profile (orange) indicates enrichment over the non-transcribed spacer. Note that, as with all repetitive regions, numbers of ChIP-seq reads mapped represent an average over all repeats. Locations of 5S rDNAs, LTRs (pink arrow) and a partial *Tcry1* retrotransposon (magenta) are indicated. Assembly of full rDNA-containing chromosome ends was not possible due to its repetitive nature, consequently the number of rDNA repeat units present at each chromosome end remains unknown. However, the total number of rDNA repeat units is estimated to be 150 from ChIP input read counts.

Source data available: GEO: GSE112454.

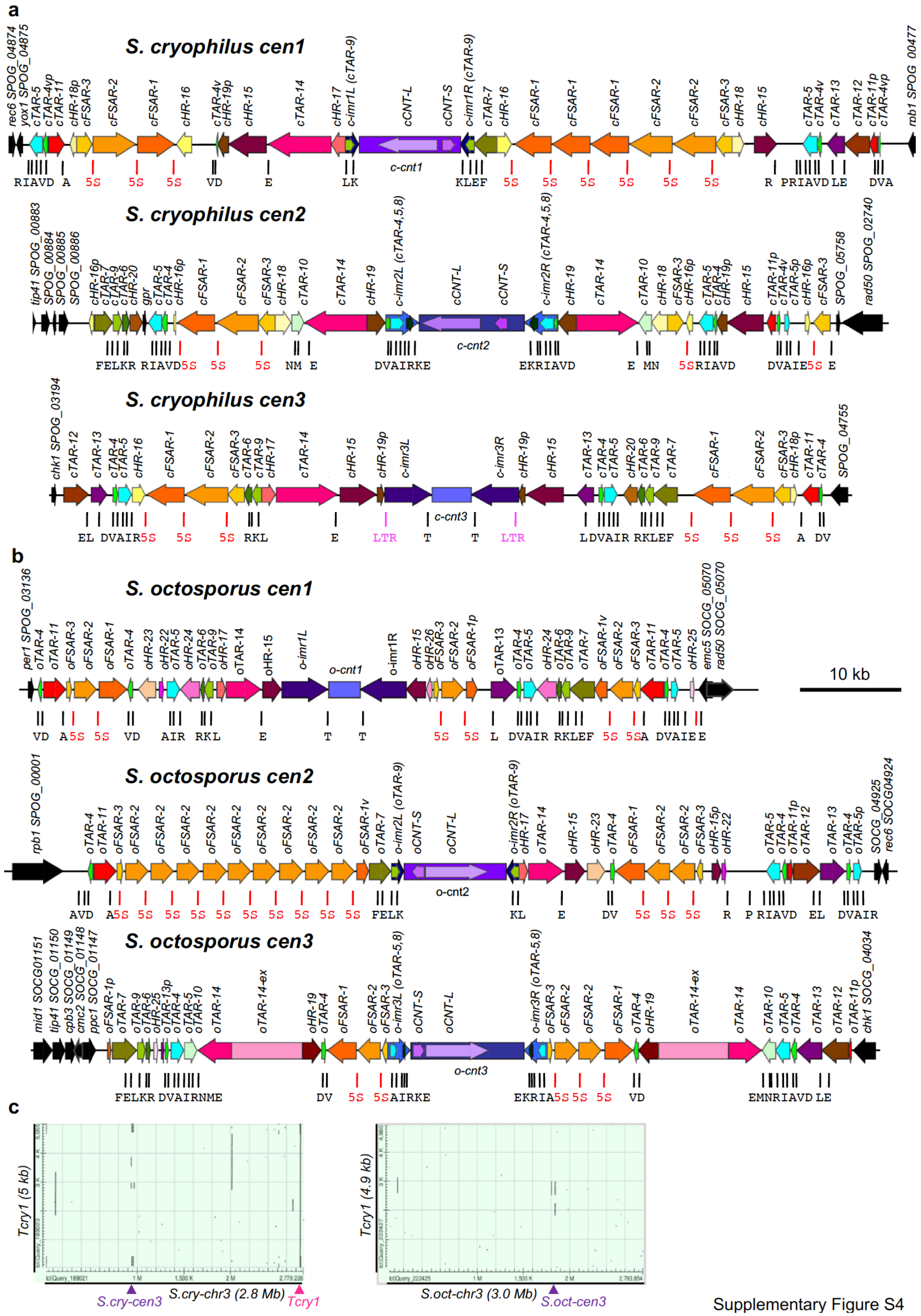

Supplementary Figure S4

Supplementary Figure 4: Centromere repeat organisation in *S. cryophilus* and *S. octosporus*
Structure and organisation of *S. cryophilus* (a) and *S. octosporus* (b) centromeres are shown. Location and names of centromeric repeats indicated. Repeat colour indicates identity/high degree of homology within species. Repeats with the same colour between species do not show sequence homology, but are present in similar contexts with respect to chromatin status, association with particular tRNA genes or occurrence and location within centromeres. The only detectable homology is between tRNA genes themselves, and between cTAR-14 (pink; all *S.cry-cens*) and extended oTAR-14-ex (*S.oct-chr3*) elements which have weak homology to the retrotransposon *Tcry1* and retrotransposon remnants at the mating-type loci of both species and at other locations in the genomes (see **Supplementary Tables 6,7,9**).
Central core regions are indicated in shades of purple, and positions of long (*cCNT-L* and *oCNT-L*) and short (*cCNT-S* and *oCNT-S*) repeats are indicated. Innermost (*imr*) inverted repeats are shown in shades of blue and in some cases contain smaller 'boundary type' repeat elements (greens). tRNA genes are shown as vertical black bars and the cognate amino acid shown in single letter code below. Small repeat elements associated with clustered tRNA genes which may have boundary function are shown in shades of green and turquoise (tRNA gene-associated repeats; TARs). 5S rDNAs are shown as vertical red bars. Heterochromatic Five-S-associated repeats (cFSARs and oFSARs) are shown in shades of orange (see **Figure 3a**). Longer repeats associated with single tRNA genes (unlikely to have boundary function) are indicated in shades of red/brown/plum (TARs 11-14). Other heterochromatic repeats (HR) are indicated in shades of yellow/brown/pink. Genes flanking the centromeres are indicated in black.
(c) Retrotransposon remnants are present within *S. cryophilus* and *S. octosporus* centromeres and other genomic locations**.** *Left panel:* Dot-plot alignment (BLASTN) of *Tcry1* retrotransposon[1] with *S. cryophilus* chromosome 3. Pink triangle indicates position of *Tcry1* itself at subtelomeric region of chr3R. Purple triangle indicates position of *S.cry-cen3*. Homology lies within cTAR-14 elements (which are also present at *S. cryophilus cen1* and *cen2*). Other regions of homology on chromosome arms are due to partial copies of *Tcry1* retrotransposon. *Right panel:* Dot-plot alignment (BLASTN) of *Tcry1* retrotransposon with *S. octosporus* chromosome 3. Purple triangle indicates position of *S.oct-cen3*. Homology within centromere is with oTAR-14ex elements, present only at *S.oct-cen3*. Region of homology in subtelomere is ~135 kb from chromosome end.
Source data available: GEO: GSE112454.

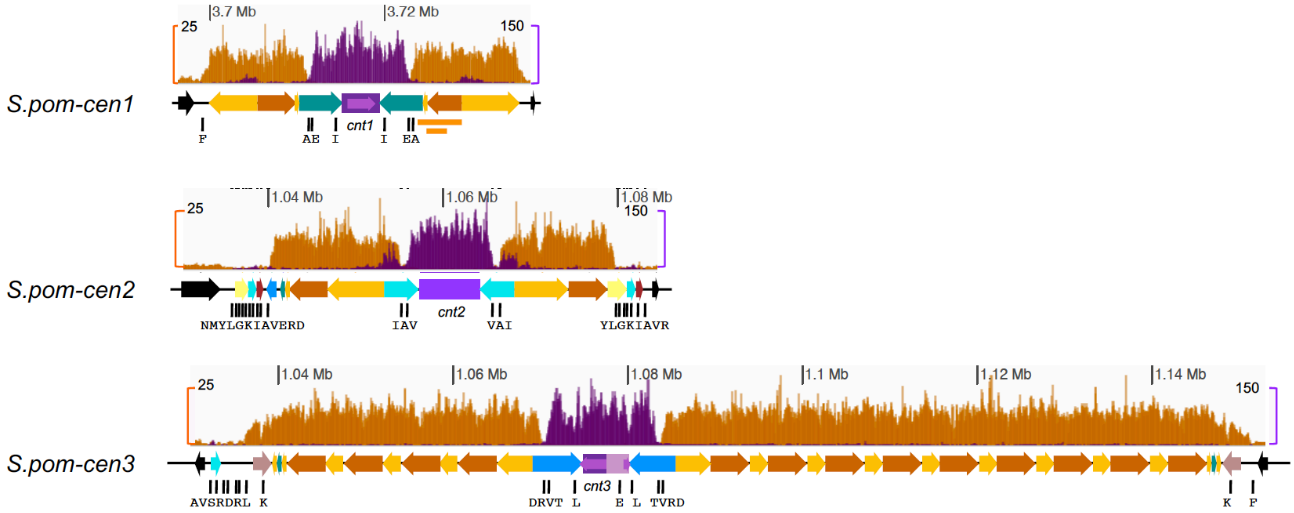

Supplementary Figure 5: Domain organisation of *S. pombe* centromeres

S. pombe genome was assembled from Oxford nanopore sequencing. *S. pombe* centromere organisation indicating repeat elements. ChIP-seq profiles for CENP-A^{Cnp1} (purple) and H3K9me2-heterochromatin (orange) are shown above each centromere. Positions of tRNA genes (single-letter code of cognate amino acid; black) are indicated. Central cores are shown in shades of purple (TM element common to *cnt1* and *cnt3* shown in mauve); innermost repeats (*imr*) in blues/greens, heterochromatic outer repeat elements are shown in dark orange (*dg*) and tangerine (*dh*). The centromeres in this strain resemble those previously described for strain SBP120390[6] rather than that of the reference genome of strain 972h^{-}[7], in the number of *K/dg/dh* repeats at *cen2* and *cen3* and in the orientation of *cnt3*. Orange bars under *S.pom-cen1* indicate the regions of heterochromatic outer-repeat present in the pK (*upper bar*) and pKp (*lower bar*) plasmids used in functional assays (see Methods).

Source data available: SRA: PRJNA472404.

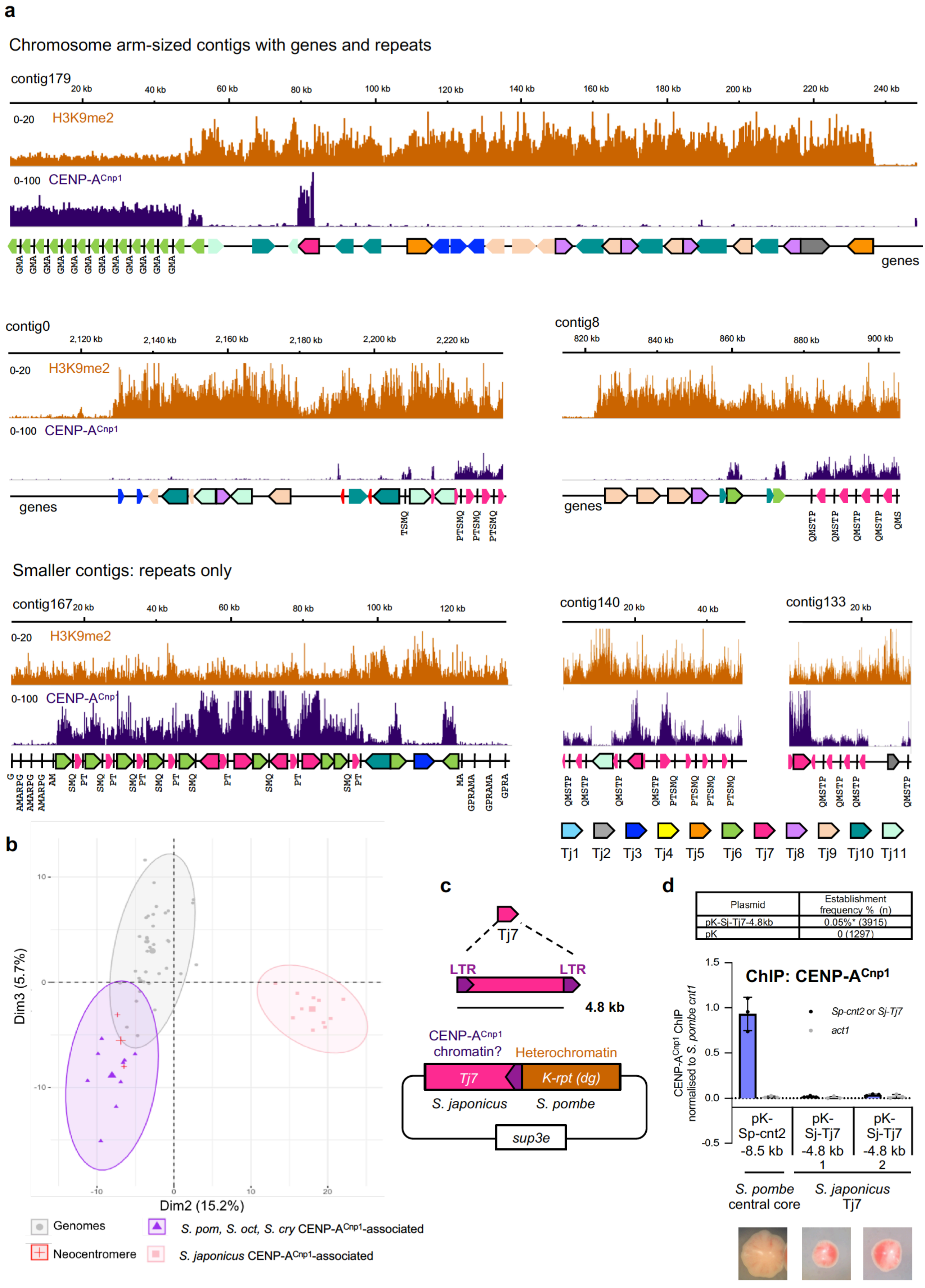

Supplementary Figure S6

Supplementary Figure 6: *S. japonicus* centromeres are rich in retrotransposons and have different underlying sequence features from other fission yeast species

(a) Representative H3K9me2 and CENP-A-associated *S. japonicus* contigs. ChIP-seq profiles for CENP-A^{Cnp1} (purple) and H3K9me2-heterochromatin (orange) are shown above each contig. Due to their repetitive nature, full assembly of centromere regions was not possible; association with CENP-A^{Cnp1} is strong indicator that a contig is centromere located. Retrotransposons[1] mapping to contigs are indicated (key, bottom), full-length or almost full-length retrotransposons are indicated by a black outline. An additional putative retrotransposon was identified which has been named Tj11 (see **Supplementary Table 4**). Positions of tRNA gene clusters (single-letter code of cognate amino acids; black) are indicated. Top/middle: Chromosome arm-sized contigs which terminate within retrotransposon arrays. Bottom: smaller contigs containing only retrotransposons and other repetitive elements could not be incorporated into genome assembly.

(b) Principal Component Analysis of 5-mer frequencies in selected sequences (12-kb window) from the four fission yeasts. PCA assigned all sequences to one of four specific groups: *S. pombe, S. cryophilus* and *S. octosporus* CENP-A^{Cnp1}-associated regions (purple); *S. japonicus* CENP-A^{Cnp1}-associated regions (pink); neocentromere forming regions (red); or other genome regions (grey). For each group the oval line encloses 95% of the data points. (See Methods for details.)

(c) The indicated region of *S. japonicus* retrotransposon Tj7 was placed adjacent to a portion of *S. pombe* heterochromatin-forming outer repeat sequence on a plasmid.

(d) Table indicates centromere establishment frequency of chimeric minichromosomes in *S. pombe* hi-CENP-A^{Cnp1} cells (n=number of transformants analysed). * 2 transformants of 3915 (0.05%) were white/pale pink upon replica plating to low adenine plates (see Methods), but they did not form sectored colonies typical of centromere establishment upon restreaking and did not assemble high levels of CENP-A^{Cnp1} (see d and Methods).

(e) *Upper*, Two candidate transformants that had potentially established centromere function on chimeric pK-Sj-Tj7 minichromosomes in *S. pombe* hi-CENP-A^{Cnp1} cells were analysed by ChIP-qPCR for CENP-A^{Cnp1}. Triplicate cultures (n=3 cultures derived from each of two transformants, i.e. technical replicates) for each transformant (1, 2) were analysed. ChIP enrichments on *S.pom-cnt2* and *S.jap-Tj7*-bearing minichromosomes (black) or a negative control locus (*act1;* grey) are normalised to the level at endogenous *S. pombe cnt1*. Individual data points are shown as black or grey dots. Error bars, standard deviation. *Lower*, *S. pombe* colonies containing pK-Sp-cnt2-8.5kb or pK-Sj-Tj7-4.8kb minichromosomes. Red colour indicates loss of minichromosome. Typical appearance: small red sectors indicate low frequency minichromosome loss and mitotic segregation function. Atypical appearance: marbled/sectored red/white colonies suggestive of poor centromere establishment.

Source data available: GEO: GSE112454 and in Source Data file.

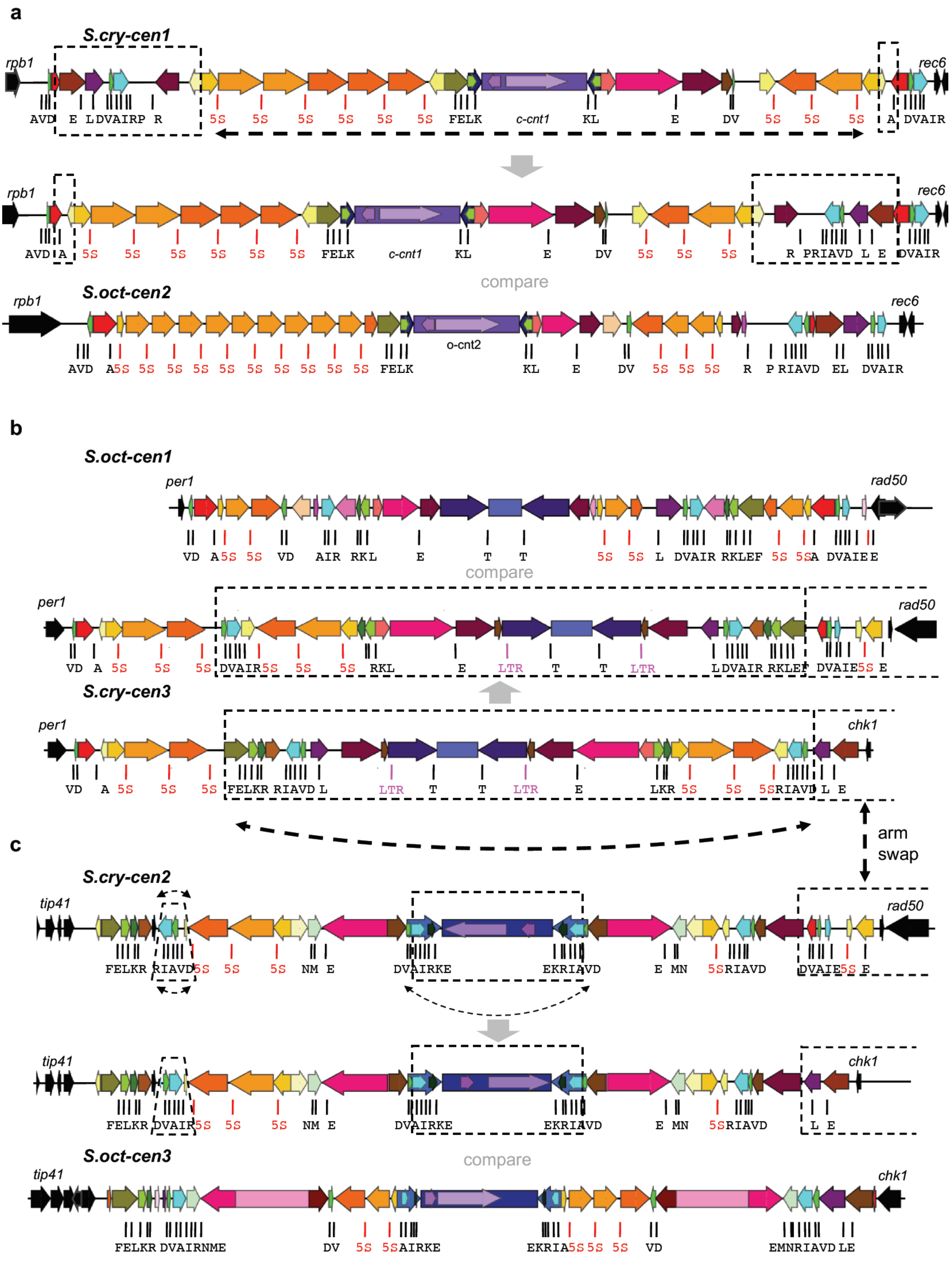

Supplementary Figure S7

Supplementary Figure 7: Synteny of tRNA gene clusters and organisation of repeat elements suggests common ancestry of *S. cryophilus* and *S. octosporus* centromeres, despite lack of sequence conservation

(a) Centromeres of *S. cryophilus* and *S. octosporus* paired with the most similar centromere from the opposite species. *In silico* rearrangements of *S. cryophilus* centromeres closely recapitulate the organisation of *S. octosporus* centromeres, providing support for their evolution from common ancestral centromeres. Dashed boxes and arrows indicate rearrangement that that would increase the structural similarity between *S.cry-cen1* and *S.oct-cen2*.

b and c) A rearrangement involving arm swap of *S.cry-cen2R* and *S.cry-cen3L* would produce synteny of genes on either side of *S.cry-cen2* and *S.cry-cen3*, and *Soct-cen3* and *Soct-cen1* respectively (centre, dashed double-headed arrow). Partial inversions (curved double-headed arrows) would increase similarity between *S. cryophilus* and *S. octosporus* centromeres (labelled compare). See **Figure 4a**.

a

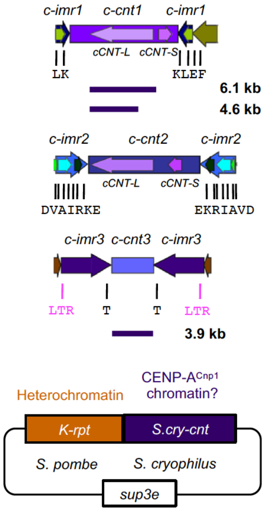

b

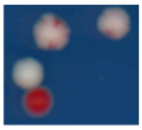

c

Plasmid	Establishment frequency % (n)	Loss rate per division % (n)
pK-Sc-cnt1-6kb	2.6 (1029)	9.4 (3784)
pK-Sc-cnt1-4.6kb	1.8 (1819)	9.6 (3788)
pKp-Sc-cnt3-3.9kb	0.2 (2280)	12.1 (4439)
pK	0 (2695)	NA

d

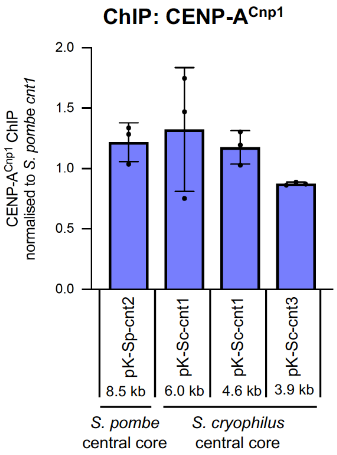

Supplementary Figure S8

Supplementary Figure 8: *S. cryophilus* central core DNA establishes CENP-A^{Cnp1} chromatin upon introduction into *S. pombe*

(a) Indicated regions of *S. cryophilus* central core DNA placed adjacent to a portion of *S. pombe* heterochromatin-forming outer repeat sequence on a plasmid.

(b) *S. pombe* cells containing pK-Sc-cnt1-6kb chimeric minichromosome were streaked to single colonies. Red colour indicates loss of minichromosome; small red sectors indicate low frequency minichromosome loss and mitotic segregation function.

(c) Establishment frequency of chimeric minichromosomes in *S. pombe* hi-CENP-A^{Cnp1} cells. Establishment frequency determined by replica plating of transformants (n=number of transformants analysed). Chromosome loss rate of established minichromosomes was determined by half-sector assay. At least two transformants containing established centromeres were analysed for each minichromosome and the mean loss rate determined, n=number of colonies screened. NA, not applicable as the minichromosomes did not establish centromere function.

(d) ChIP-qPCR for CENP-A^{Cnp1} on *S. pombe* hi-CENP-A^{Cnp1} cells containing chimeric minichromosomes with established centromere function. Three biologically independent transformants were analysed for each minichromosome (n=3). ChIP enrichment on *S.pom-cnt2* and *S.cry-cnt*-bearing minichromosomes is normalised to the level at endogenous *S. pombe cnt1*. Individual data points are shown as black dots. Error bars, standard deviation.

Source data available as a Source Data file.

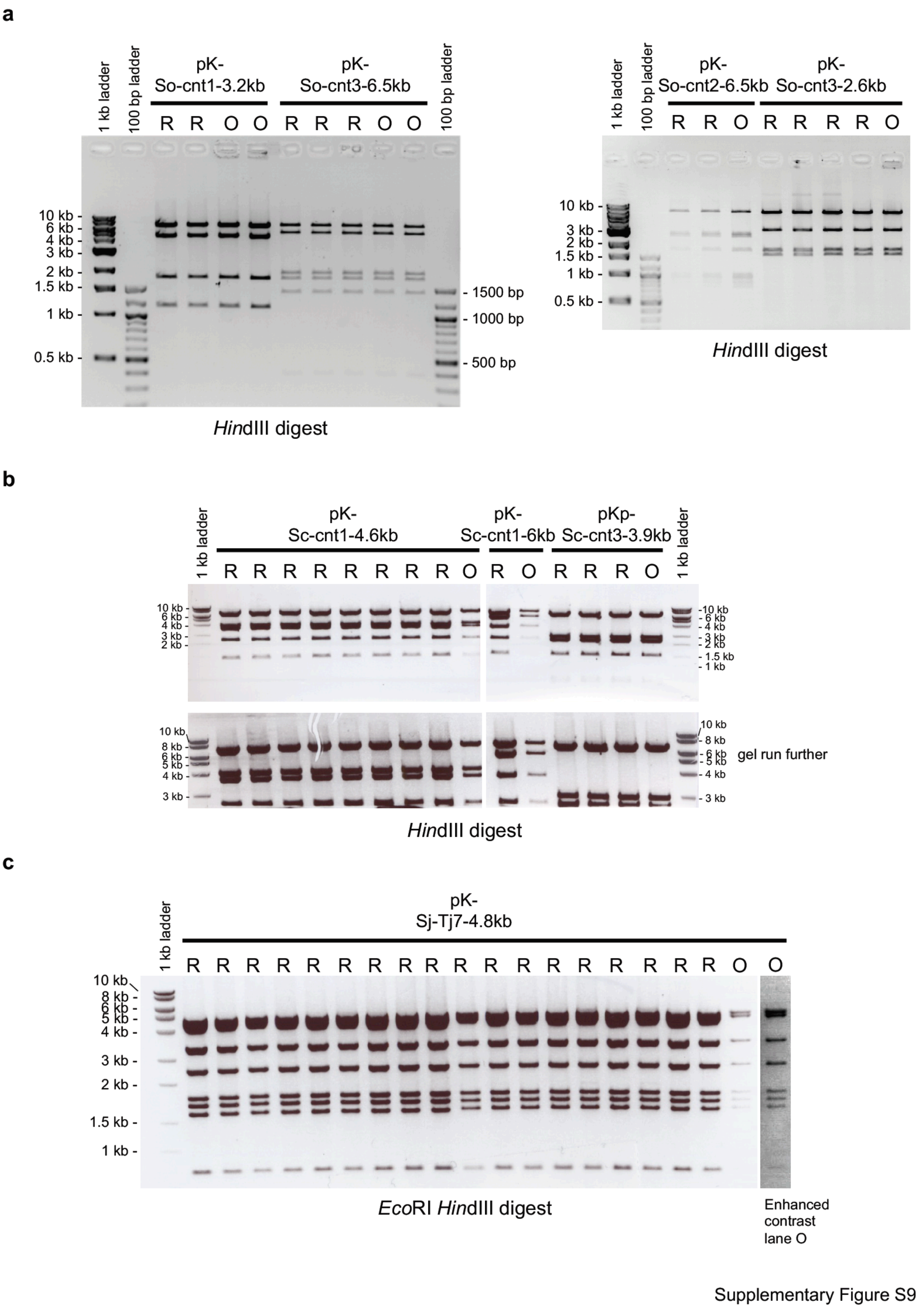

Supplementary Figure 9: Minichromosomes remain intact in *S. pombe*

(a) Minichromosomes bearing *S. octosporus* central core DNA do not become rearranged nor acquire host DNA in *S. pombe*. Indicated minichromosomes with established centromere function were isolated from *S. pombe* and recovered in *E. coli*. DNA was digested with *Hin*dIII and run on an agarose gel containing ethidium bromide. Recovered plasmids (R), are run alongside original plasmids (O) for comparison.

(b) Minichromosomes bearing *S. cryophilus* central core DNA do not become rearranged nor acquire host DNA in *S. pombe*. *Hin*dIII digest. Analysis as in (a).

(c) Minichromosomes bearing *S. japonicus Tj7* DNA do not become rearranged nor acquire host DNA in *S. pombe*. *Eco*RI *Hin*dIII digest. Analysis as in (a).

Source data available as a Source Data file.

Supplementary References

1. Rhind, N. *et al.* Comparative functional genomics of the fission yeasts. *Science* **332,** 930–936 (2011).
2. Ács-Szabó, L., Papp, L.A., Antunovics, Z., Sipiczki, M., & Miklós I. Assembly of *Schizosaccharomyces cryophilus* chromosomes and their comparative genomic analyses revealed principles of genome evolution of the haploid fission yeasts. *Sci. Rep.* **8**,14629 (2018).
3. Noma, K.-I., Cam, H. P., Maraia, R. J. & Grewal, S. I. S. A role for TFIIIC transcription factor complex in genome organization. *Cell* **125,** 859–872 (2006).
4. Sanchez, J. A., Kim, S. M. & Huberman, J. A. Ribosomal DNA replication in the fission yeast, *Schizosaccharomyces pombe*. *Experimental Cell Research* **238,** 220–230 (1998).
5. Tashiro, S., Nishihara, Y., Kugou, K., Ohta, K. & Kanoh, J. Subtelomeres constitute a safeguard for gene expression and chromosome homeostasis. *Nucleic Acids Res* **45,** 10333–10349 (2017).
6. Steiner, N. C., Hahnenberger, K. M. & Clarke, L. Centromeres of the fission yeast Schizosaccharomyces pombe are highly variable genetic loci. *Mol Cell Biol* **13,** 4578–4587 (1993).
7. Wood, V. *et al.* The genome sequence of *Schizosaccharomyces pombe*. *Nature* **415,** 871–880 (2002).
