# Supplementary material for: Interspecies conservation of organisation and function between nonhomologous regional centromeres
Source: Nat Commun. 2019 May 28;10:2343. doi: 10.1038/s41467-019-09824-4 (PMC6538654; doi:10.1038/s41467-019-09824-4)
Supplement: Supplementary file 3 — Description of Additional Supplementary Files [file 41467_2019_9824_MOESM3_ESM.pdf]

Description of Additional Supplementary Files

File Name: Supplementary Data 1
Description: *S. cryophilus* telomere and subtelomere repeat co-ordinates

File Name: Supplementary Data 2
Description: *S. octosporu*s telomere and subtelomere repeat co-ordinates

File Name: Supplementary Data 3
Description: *S. cryophilus* centromere repeat co-ordinates

File Name: Supplementary Data 4
Description: *S. octosporus* centromere repeat co-ordinates

File Name: Supplementary Data 5
Description: *S. pombe* centromere repeat co-ordinates

File Name: Supplementary Data 6
Description: *S. cryophilus* rDNA co-ordinates

File Name: Supplementary Data 7
Description: *S. octosporus* rDNA co-ordinates

File Name: Supplementary Data 8
Description: *S. cryophilus* and *S. octosporus* hsp16 ORFs

File Name: Supplementary Data 9
Description: *S. cryophilus* tDNA co-ordinates

File Name: Supplementary Data 10
Description: *S. octosporus* tDNA co-ordinates

File Name: Supplementary Data 11
Description: *S. japonicus* CENP-A associated regions: co-ordinates for PCA (Supp Fig 6)
