# Supplementary material for: Interspecies conservation of organisation and function between nonhomologous regional centromeres
Source: Nat Commun. 2019 May 28;10:2343. doi: 10.1038/s41467-019-09824-4 (PMC6538654; doi:10.1038/s41467-019-09824-4)
Supplement: Supplementary file 15 — Reporting Summary [file 41467_2019_9824_MOESM15_ESM.pdf]

nature research

Corresponding author(s): Robin Allshire & Alison Pidoux

Reporting Summary

Nature Research wishes to improve the reproducibility of the work that we publish. This form provides structure for consistency and transparency in reporting. For further information on Nature Research policies, see Authors & Referees and the Editorial Policy Checklist.

Statistical parameters

When statistical analyses are reported, confirm that the following items are present in the relevant location (e.g. figure legend, table legend, main text, or Methods section).

n/a	Confirmed	
☐	☒	The exact sample size (n) for each experimental group/condition, given as a discrete number and unit of measurement
☐	☒	An indication of whether measurements were taken from distinct samples or whether the same sample was measured repeatedly
☐	☒	The statistical test(s) used AND whether they are one- or two-sided<br>*Only common tests should be described solely by name; describe more complex techniques in the Methods section.*
☐	☒	A description of all covariates tested
☐	☒	A description of any assumptions or corrections, such as tests of normality and adjustment for multiple comparisons
☐	☒	A full description of the statistics including central tendency (e.g. means) or other basic estimates (e.g. regression coefficient) AND variation (e.g. standard deviation) or associated estimates of uncertainty (e.g. confidence intervals)
☐	☒	For null hypothesis testing, the test statistic (e.g. F, t, r) with confidence intervals, effect sizes, degrees of freedom and P value noted<br>*Give P values as exact values whenever suitable.*
☒	☐	For Bayesian analysis, information on the choice of priors and Markov chain Monte Carlo settings
☒	☐	For hierarchical and complex designs, identification of the appropriate level for tests and full reporting of outcomes
☐	☒	Estimates of effect sizes (e.g. Cohen's d, Pearson's r), indicating how they were calculated
☐	☒	Clearly defined error bars<br>*State explicitly what error bars represent (e.g. SD, SE, CI)*

Our web collection on statistics for biologists may be useful.

Software and code

Policy information about availability of computer code

Data collection	SMRT portal 2.0;<br>HGAP version 3 ;<br>Bowtie2 version 2.2.6;<br>Samtools version 1.3.1;<br>Deeptools version 3.0.0;<br>MACS2 version 2.1.1;<br>IGViewer;<br>Liftover (UCSC: http://hgdownload.soe.ucsc.edu/downloads.html#source_downloads);<br>Crossmap;<br>MUMmer3.23;<br>blastall version 2.2.26<br>tRNAscan-SE-1.3.1;<br>Dfam 2.0;<br>BEAST v1.8.4 and BEAUti v1.8.4<br>FigTree v1.4.3
Data analysis	SMRT portal 2.0 and HGAP version 3 were use for assemble the new genomes;<br>Liftover from UCSC, Crossmap, MUMmer3.23, blastall version 2.2.26, tRNAscan-SE-1.3.1 and Dfam 2.0 were used to annotated the assembled genome sequence.

For manuscripts utilizing custom algorithms or software that are central to the research but not yet described in published literature, software must be made available to editors/reviewers upon request. We strongly encourage code deposition in a community repository (e.g. GitHub). See the Nature Research guidelines for submitting code & software for further information.

Data

Policy information about availability of data

All manuscripts must include a data availability statement. This statement should provide the following information, where applicable:
- Accession codes, unique identifiers, or web links for publicly available datasets
- A list of figures that have associated raw data
- A description of any restrictions on data availability

GSE112454; PRJNA472404

Field-specific reporting

Please select the best fit for your research. If you are not sure, read the appropriate sections before making your selection.

☒ Life sciences ☐ Behavioural & social sciences

For a reference copy of the document with all sections, see nature.com/authors/policies/ReportingSummary-flat.pdf

Life sciences

Study design

All studies must disclose on these points even when the disclosure is negative.

Sample size	ChIP-seq: 2 biological replicates for each S. octosporus and S. cryophilus CENP-A and H3K9me2 ChIP-seq, and S. pombe H3K9me2 ChIP-seq. 1 ChIP-seq was performed for each of S. pombe CENP-A, S. japonicus CENP-A and H3K9me2.<br>3 independent biological replicates were used for ChIP-qPCR.<br>2 biological replicates were used for determination of chromosome loss frequency. Numbers of colonies counted are indicated.
Data exclusions	There were no data exclusions.
Replication	Findings were reliably reproduced.
Randomization	Samples were not randomised.
Blinding	Experiments were conducted in unblinded conditions.

Materials & experimental systems

Policy information about availability of materials

n/a	Involved in the study
☒	☐ Unique materials
☐	☒ Antibodies
☒	☐ Eukaryotic cell lines
☒	☐ Research animals
☒	☐ Human research participants

Antibodies

Antibodies used	mouse mAb 5.1.1 H3K9me2; sheep CENP-A-Cnp1 antiserum
Validation	mouse mAb 5.1.1: Raised in Urano Lab, Nakagawachi et al (2003) Oncogene 22, 8835<br>sheep anti-CENP-A-Cnp1: raised in Allshire Lab, Castillo et al (2007) PLoS Genet. 3, e121

Method-specific reporting

n/a	Involved in the study
☐	☒ ChIP-seq
☒	☐ Flow cytometry
☒	☐ Magnetic resonance imaging

ChIP-seq

Data deposition

☒ Confirm that both raw and final processed data have been deposited in a public database such as GEO.

☒ Confirm that you have deposited or provided access to graph files (e.g. BED files) for the called peaks.

Data access links
May remain private before publication.

GSE112454
PRJNA472404

Files in database submission

cry_CENPA_PE
cry_CENPA_SE
cry_H3K9_PE
cry_H3K9_RA_PE
cry_INPUT
cry_input_PE
cry_input_RA_PE
jap_cenpa_SE
jap_H3K9_SE
jap_INPUT
oct_CENPA_PE
oct_CENPA_SE
oct_H3K9_PE
oct_H3K9_RA_PE
oct_H3K9_SE
oct_input_PE
oct_input_RA_PE
oct_INPUT_SE
pombe_cenpa_PE
pombe_H3K9_RA_PE
pombe_h90-1_H3K9_PE
pombe_h90-1_input_PE
pombe_h90-2_H3K9_PE
pombe_h90-2_INPUT_PE
pombe_input_PE
pombe_input_RA_PE

Genome browser session
(e.g. UCSC)

https://www.ncbi.nlm.nih.gov/geo/query/acc.cgi?acc=GSE112454

Methodology

Replicates

2 biological replicates for each S. octosporus and S. cryophilus CENP-A and H3K9me2 ChIP-seq, and S. pombe H3K9me2 ChIP-seq. 1 ChIP-seq was performed for each of S. pombe CENP-A, S. japonicus CENP-A and H3K9me2.
cry_CENPA 2 replicates 0.975
CRY_h3k9 2 replicates 0.884
oct_CENPA 2 replicates 0.954
oct_H3K9 2 replicates 0.917
pombe_h90_H3k9 2 replicates 0.991
Wild-type yeast strains growing in standard conditions were used. No experimental conditions were applied.

Sequencing depth

sample total mapped length type
cry_CENPA_PE 7711564 6584772 100 PE
cry_H3K9_PE 14590750 4059485 100 PE
cry_input_PE 10976896 10447542 100 PE
oct_CENPA_PE 6551994 5569244 100 PE
oct_input_PE 13720042 13354900 100 PE
pombe_cenpa_PE 3693812 3512811 100 PE
pombe_input_PE 11607092 11185468 100 PE
cry_H3K9_RA_PE 13751978 5064753 50 PE
cry_input_RA_PE 24035122 23595971 50 PE
oct_H3K9_RA_PE 23947410 17425639 50 PE
oct_input_RA_PE 18137784 10785346 50 PE
pombe_H3K9_RA_PE 11861370 7895248 50 PE

pombe_input_RA_PE 26938324 26519498 50 PE
pombe_h90-1_H3K9_PE 17044938 8003083 76 PE
pombe_h90-1_input_PE 5680886 5578302 76 PE
pombe_h90-2_H3K9_PE 16531684 8586065 76 PE
pombe_h90-2_INPUT_PE 6431868 6304943 76 PE
cry_CENPA_SE 3542090 3459719 49 SE
cry_INPUT 3578650 3565119 49 SE
jap_cenpa_SE 3573436 3436191 49 SE
jap_H3K9_SE 3581979 2916219 49 SE
jap_INPUT 3576275 3338528 49 SE
oct_CENPA_SE 3555717 3515159 49 SE
oct_H3K9_SE 3550612 2977985 49 SE
oct_INPUT_SE 3554467 3543059 49 SE
cd36Cnp1 7698217 7468972 50 SE
cd60Cnp1 4988732 4437050 50 SE

replicates
cry_CENPA 2 replicates 0.975
CRY_h3K9 2 replicates 0.884
oct_CENPA 2 replicates 0.954
oct_H3K9 2 replicates 0.917
pombe_h90_H3k9 2 replicates 0.991

Antibodies

mouse mAb 5.1.1 H3K9me2; sheep CENP-A-Cnp1 antiserum;

Peak calling parameters

macs2 callpeak -g -f BAMPE -t -c --ratio RATIO

Data quality

MACS2 was used to call peaks from Paired-end ChIP-seq data.

Software

Bowtie2 version 2.2.6;
Samtools version 1.3.1;
Deeptools version 3.0.0;
MACS2 version 2.1.1;
IGViewer;
